# Perioperative Patient Blood Management in Primary Knee and Hip Arthroplasty—Nonsense or Necessity?

**DOI:** 10.3390/jcm14228237

**Published:** 2025-11-20

**Authors:** Johannes Neugebauer, Clemens Strassegger, David Putzer, Melanie Schindler, Adriana Palacio-Giraldo, Markus Neubauer, Gianpaolo Leone, Herbert Koinig, Dietmar Dammerer

**Affiliations:** 1 Karl Landsteiner University of Health Sciences, Dr. Karl-Dorrek-Straße 30, 3500 Krems, Austria; clemens.strassegger@baden.lknoe.at (C.S.); melanie.schindler@krems.lknoe.at (M.S.); adriana.palaciogiraldo@krems.lknoe.at (A.P.-G.); markus.neubauer@krems.lknoe.at (M.N.); herbert.koinig@krems.lknoe.at (H.K.); dietmar.dammerer@krems.lknoe.at (D.D.); 2 Devison of Orthopedics and Traumatologie, University Hospital Krems, Mitterweg 10, 3500 Krems, Austria; gianpaolo.leone@krems.lknoe.at; 3 Danube University Krems, University for Continuing Education, Dr. Karl-Dorrek-Straße 30, 3500 Krems, Austria; 4 Department of Orthopedics and Traumatologie, Experimental Orthopaedics, Medical University Innsbruck, Anichstrasse 35, 6020 Innsbruck, Austria; david.putzer@i-med.ac.at

**Keywords:** patient blood management, transfusion, anemia, preoperative patient management

## Abstract

**Background/Objectives:** The perioperative management of a patient’s blood values involves the optimization of anemia management in the pre-, intra-, and postoperative periods to avoid blood transfusions. The purpose of this study was to determine the prevalence of pre- and postoperative anemia in patients undergoing elective knee and hip arthroplasty, and explore the change in hemoglobin levels in transfused and non-transfused patients over the course of their hospital stay. **Methods**: In this retrospective study, data on anemia-influencing factors were collected and examined using multiple regression analysis (OLS model) to determine if they are associated with the average hemoglobin change in 801 patients who underwent a primary knee or hip arthroplasty. The study group was screened for the prevalence of preoperative anemia, and the incidence of postoperative anemia was examined. **Results**: A total of 801 patients were retrospectively investigated. The length of stay for the two different procedures was significantly different (*p* < 0.001). There was a markedly higher transfusion rate in the cases requiring longer-than-usual hospital stays. In total, 37 patients (5%) received perioperative erythrocyte transfusions. Total hip replacement (THR) was associated with significantly more erythrocyte transfusions (8%) in comparison with total knee replacement (TKR) (2%) (*p* < 0.001). **Conclusions**: Satisfactory results for the prevalence and incidence of anemia in the context of primary knee or hip arthroplasties were obtained in this study. The collected values indicate the need for better implementation and awareness of the importance of adequate patient blood management.

## Simple Summary

The increase in arthroplasties has led to a focus on PBM. To avoid non-indicated blood transfusions, particularly red blood cell concentrates, this study was performed to determine the prevalence of pre- and postoperative anemia in patients undergoing primary knee and hip replacement surgery. Therefore, we investigated our own perioperative anemia management with a focus on the factors that can be optimized preoperatively.

## 1. Introduction

The number of hip and knee arthroplasties has reached a new high; according to the 2023 annual report of the German Endoprosthesis Register (EPRD), 378.812 hip and knee arthroplasty procedures were performed in 2023. The current literature suggests that from 2005 to 2030, the number of THRs will increase by 174% to nearly 600,000, and the situation with TKRs will be similar [1]. The number of transfusions is also steadily increasing worldwide, including those for orthopedic and trauma surgeries [2]. At the same time, blood is becoming a scarce resource [2]. The average blood loss during primary joint arthroplasty is 650 mL [2] and the probability of a primary joint arthroplasty requiring a transfusion is 1–10% [2].

Anemia is defined as a decreased concentration of hemoglobin, a reduced number of red blood cells, or a hematocrit below a defined limit [3,4]. It can be congenital or acquired [3,4,5,6,7]. The hemoglobin level thresholds (in g/dL) to diagnose anemia at sea level are outlined by the WHO’s “Hemoglobin concentrations for the diagnosis of anemia and assessment of severity 2011” [8]. The classification of anemia is based on erythrocyte indices such as MCV, MCH, and MCHC (normo-, micro-, and macrocytic) and Hb concentration (normo-, hypo-, and hyperchromic) [5,6]. The treatment is based on the underlying cause [9].

Anemia of chronic disease (ACD) is characterized by a reduced ferritin (Fe) concentration, although the level remains in the normal range. The pro-inflammatory cytokine IL-6, hepcidin (a membrane protein), and the Fe exporter IREG-1 cause a decrease in the production of red blood cells (RBCs), resulting in anemia. Different from Fe deficiency, anemic patients have normal Fe levels and the coexistence of both conditions is possible. Chronic infections and chronic inflammatory and neoplastic diseases are the main causes of ACD. ACD can be diagnosed using other parameters such as a low erythropoietin (EPO) level and an increased C-reactive protein (CRP) level. A direct treatment (i.e., blood transfusion) is only necessary in the case of severe anemia [10,11,12]. Obesity is highly associated with increased levels of pro-inflammatory cytokines, such as IL-6, which stimulate the production of hepcidin in adipocytes and hepatocytes. This may lead to functional Fe deficiency [11,13,14].

Iron deficiency anemia occurs due to a low whole-body Fe content (50–170 mcg/dL in females; 65–175 mcg/dL in males). In chronic Fe deficiency or hemorrhage, the storage of iron as ferritin decreases first (to 12–150 ng/mL in females and 15–200 ng/mL in males), followed by decreases in serum iron and transferrin (220–400 mg/dL). This leads to low hemoglobin levels and a low hematocrit [7,15]. There are many causes of iron deficiency anemia [7]: acute and chronic hemorrhaging, reduced iron ingestion (e.g., due to vegetarian or vegan diets or young age), malabsorption (due to stomach surgery, diseases of the duodenum, or pharmaceuticals), or an increased iron demand (e.g., during pregnancy).

Patients with renal anemia have a normochromic, normocytic blood count but fewer reticulocytes (<30,000/µL). Further laboratory analysis, especially the measurement of creatinine levels, (e) GFR, and urea levels, is also important for a diagnosis. Renal anemia is classified as a decreased synthesis of EPO due to chronic kidney failure. However, it should be noted that low reticulocyte counts can be caused by numerous other etiologies (e.g., iron deficiency, vitamin B12 deficiency, aplastic anemia). Therefore, if renal anemia is suspected, the other etiologies must be ruled out and an interdisciplinary differential diagnosis should be established prior to a renal anemia diagnosis [16].

Preoperative anemia has a prevalence of 30–50% across Europe [2,9,17,18,19,20]. It is one of the most important risk factors for postsurgical complications and mortality, especially among older patients, and is one of the strongest predictors for the administration of red blood cell concentrates [2,9,17,21,22,23,24]. Patients with mild anemia have a 30% higher relative risk of complications and death [9]. Therefore, a precise diagnosis and treatment are highly recommended in elective surgery [2,17,22,23,24]. The optimal use of blood components can improve the course of the disease and significantly relieve strained hospital budgets [5,6,17,25,26,27]. Before elective operations with an expected perioperative blood loss greater than 500 to 1000 mL, it is recommended that the wait time for surgery should be used to establish a differential diagnosis of preoperative anemia and treat the causes. If the wait time is too short and it is medically justifiable, the procedure should be postponed by two to eight weeks [5,6]. Compared with allogenic blood transfusion, preoperative treatment of anemia is more economically beneficial as it helps to relieve the issue of general scarcity of blood products and leads to better outcomes and less transfusion-associated risks [5].

The prehistory, accompanying illnesses, symptoms, and hemoglobin concentration (grams of hemoglobin per deciliter of blood, g/dL) of the patient are used to determine the need for erythrocyte transfusion [28]. Cut-off values in the literature range from 6 to 10 g/dL [28]. The physiologic transfusion triggers are (a) cardiopulmonary symptoms (tachycardia, hypotension, a decline in blood pressure without another plausible cause, or dyspnea); (b) echocardiography (ECG) findings typical of ischemia (newly occurring ST elevations or depressions, arrythmias, or region of impaired myocardial contractility); and (c) global indices of insufficient oxygen delivery (increase in global oxygen extraction to >50%, decrease in oxygen uptake to >10% of initial value, decrease in mixed venous oxygen saturation to <50%, decrease in mixed venous pO_2_ to <32 mmHg, decrease in central venous oxygen saturation to <60%, or lactic acidosis (lactate > 2 mmol/L and blood pH < 7.35)) [28,29].

Other influencing factors are age, gender, comorbidities, acute versus chronic bleeding, severity of anemia, individual anemia tolerance, and risk of further bleeding [3,4,28,30]. All these factors may lead to a decreased individual anemia tolerance and subsequently to an increased critical hemoglobin concentration (Hb_crit_), i.e., the minimum hemoglobin concentration a certain individual can tolerate [28]. Women’s hemoglobin concentration and their tolerance to anemia are, on average, 12% less than that of age- and ethnicity-matched men [31,32,33]. Most guidelines do not account for sex-specific transfusion triggers or transfusion volumes [34,35].

Furthermore, there are organ-specific oxygen demands depending on the patient’s metabolic activity and physical activity [36]. For example, the demand can increase up to 15-fold during exercise [36]. The anemia tolerance of various organs can be measured through the quantification of pimonidazole–protein adducts, vascular endothelial growth factor (VEGF) and mRNA expression, and markers for tissue hypoxia after severe hemodilution [30]. The kidneys have a particularly low anemia tolerance and can show molecular signs of tissue hypoxia at systemic oxygen supply levels and hemoglobin-based transfusion triggers well above the critical limits. In contrast, the heart, liver, and brain often show no signs of hypoxia before the critical hemoglobin concentration is reached [30]. Therefore, signs of acute kidney injury, like oliguria or anuria, are to be expected during periods of prolonged anemia.

Initial blood sampling (measurements of complete blood count, erythrocyte indices, iron status, and vitamin B12 and folate levels, among others) may give important insights into how much time is needed before the patient can be in their best hematologic state. During the surgical procedure, minimally invasive and blood-sparing techniques (e.g., smaller surgical incisions, intraoperative cell salvage), as well as cautious and vigilant hemostatic measures should be used whenever possible. Additional actions, such as inducing hypotension or low venous pressure, can also be beneficial. Hemostatic drugs such as tranexamic acid (an antifibrinolytic) and desmopressin (a von Willebrand factor-releasing drug) have also shown effectiveness in hemorrhage prophylaxis [5,6,37,38]. The patient’s hemostatic capability should be carefully monitored as well, using point-of-care testing [5,6,39]. Lastly, the patients’ physiologic reserves should be exploited as much as possible, oxygen delivery should be maximized alongside minimized oxygen consumption, and restrictive transfusion protocols should be implemented and adhered to if medically possible [5,6].

After the procedure is performed, iatrogenic blood loss (from diagnostic blood tests) should be minimized [40] as the overall diagnostic blood loss is believed to be much too high in most hospitals [41]. If necessary, prophylactic iron administration and erythropoiesis-stimulating agents should be started, and impaired hemostasis should be corrected as soon as possible [37,39].

The purpose of the present study was to determine the prevalence of pre- and postoperative anemia in patients undergoing primary knee and hip arthroplasty in an elective setting and to explore the changed in hemoglobin levels in transfused and non-transfused patients over the course of their hospital stay.

## 2. Material and Methods

The study was approved by the Commission for Scientific Integrity and Ethics of the Karl Landsteiner Private University of Health Sciences in Krems an der Donau, Austria (EK Nr: 1037/2021). This study involved retrospective observational data analysis. All the patients that underwent either an elective primary total knee replacement (TKR) (including unicondylar knee arthroplasty) or an elective primary total hip replacement (THR) from 1 July 2018 to 26 December 2019 at the Department of Orthopedics and Traumatology at the University Hospital Krems, Austria, were included in the study. The inclusion criteria were (a) an elective primary total knee replacement (including unicondylar knee arthroplasty) or an elective primary total hip replacement; (b) preoperative laboratory results for blood counts, electrolytes, the kidney panel, and the liver panel; and (c) one postoperative laboratory investigation including the above-mentioned parameters. These investigations could be performed either at laboratory institutes outside of the hospital or at the hospital itself. The patients that received an arthroplasty due to an emergency procedure, e.g., a fracture situation, were excluded because these cases may not represent standard transfusion practice. Patients younger than 18 years or older than 99 years were also excluded. A total of 846 patients were identified. Another 45 patients had to be excluded due to the above-mentioned exclusion criteria or because they had incomplete data sets. Therefore, our study group consisted of 801 patients. The laboratory data were acquired at two time points: (a) at admission to the hospital (either the earliest preoperative blood sampling performed within the hospital or preoperative lab reports performed at a private practice) and (b) at “discharge” (the last postoperative lab investigation performed during the same hospital stay). Patient data were collected and analyzed to determine the prevailing anemia and extent of transfusion of erythrocyte concentrates. Anemia-relevant blood parameters were collected: RBC count, Hb level, Hct level, MCH, MCV, and MCHC. In this study, we used the RBC count and Hct and Hb levels to determine the type of blood anemia. The hemoglobin reference values used to diagnose anemia were obtained from the WHO’s publication “Hemoglobin concentrations for the diagnosis of anemia and assessment of severity 2011” [8]. A Hb level below <7.0 was considered severe anemia, between 7.0 and 9.9 was defined as moderate anemia, between 10.0 and 10.9 was considered mild anemia, and above ≥11.0 was defined as non-anemic.

During the acquisition process, the data was collected using Microsoft^®^ Excel for Mac^®^ (version 16.51; Microsoft Corporation, Redmond, CA, USA). For the statistical analysis, GraphPad Prism (version 10.2.3; GraphPad Software LLC., Boston, MA, USA) was used. Normal distribution was tested using the Shapiro–Wilk Test when the sample size was >50 and Kolmogorov–Smirnov Test if the sample size was <50 for each variable. QQ plots were plotted and visually inspected.

A *p*-value of 0.05 was considered statistically significant. The Mann–Whitney U-Test was used for non-normally distributed variables with independent samples and for continuous data. It was used to compare the length of stay (LOS) between the TKR and THR groups and to compare female and male patients’ RBC counts and Hb and Hct levels at admission and the Hb levels of patients who received or did not receive a transfusion. The Mann–Whitney U test was also used to compare the changes in Hb levels of the groups based on the type of surgery and gender. The Wilcoxon matched-pairs signed-rank test was used to evaluate sex-specific differences (not normally distributed) in Hb levels at admission and discharge. There were no missing values for the Hb levels at admission and discharge. Effect size was calculated for the Mann–Whitney U test and for the Wilcoxon matched-pairs signed-rank test using the rank biserial correlation, which is more suitable for non-normally distributed data. Fisher’s exact test was used to compare the need for erythrocyte transfusion of the different groups as the sample size was small. The effect size was reported as the relative risk (RR). Kruskal–Wallis with Dunn’s multiple comparison post hoc test with Šidák correction was used to compare the Hb levels of different age groups, as well as the changes in RBC count and Hb and Htc levels of different age and LOS groups. Effect size was calculated using the ε^2^ method. Each *p*-value obtained from the pairwise comparisons was adjusted to account for multiple comparisons, and the effect size was calculated using Cohen’s r. The Chi^2^ test was used to compare the admission and discharge data of the patients at each anemia severity level (severe, moderate, mild, and non-anemic) as there were more than 2 classes. Effect size was calculated using Cramér’s V. All boxplots show the median and 25th and 75th quartiles (box), as well as the minimum and maximum values (whiskers).

## 3. Results

**Figure 1:**
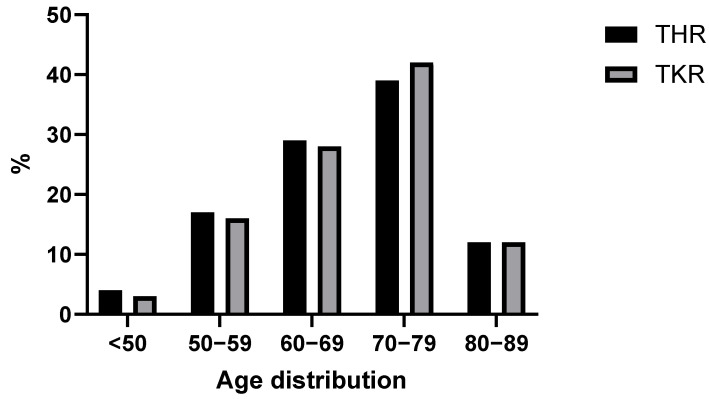
Distribution of patients by age group.

A total of 801 patients (female: 481; male: 320) were retrospectively investigated. The study group consists of 354 THR patients (female: 200; male: 154) and 447 TKR patients (female: 281; male: 166) (see Table 1). Among the women, 200 (42%) underwent THR and 281 (58%) underwent TKR. Among the men, 154 (48%) underwent THR and 166 (52%) underwent TKR (Table 1). The median age was 70 years (IQR 15); the oldest patient was 89 and the youngest was 23 years old (Figure 1). The largest subpopulation by age were 70–80-year-old patients with a population size of 332 individuals (~40%). There were 27 (~3%) patients younger than 50.

**Figure 2:**
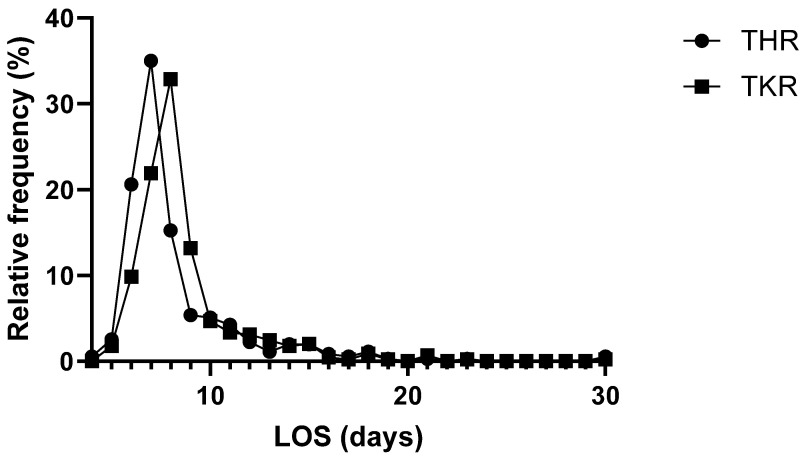
Distribution of durations of hospital stay in THR and TKR groups. Each point represents a 2-day bin.

The average patient stayed for eight days (median) at the hospital (Table 1). The shortest stay was four days, whereas the longest stay was 90 days. A total of 559 patients (~70%) stayed between 4 and 8 days in the hospital, 223 (~28%) stayed between 9 and 17 days, and 19 (~2%) stayed longer than 17 days in the hospital (Figure 2). Three patients were admitted to the intensive care unit (ICU) after surgery; one of them (<1%) stayed for 2 days, one stayed for 4 days, and one stayed for 12 days. The length of stay (LOS) of patients receiving THR was 7 days (mean: 8; median: 7; Q1: 7; Q2: 9; IQR: 2; min: 4; max: 30); for TKR patients, the LOS was 8 days (mean: 9; median: 8; Q1: 7; Q2: 9; IQR: 2; min: 5; max: 30). The LOSs of the TKR and THR groups showed a normal distribution (both *p* < 0.001) and showed a statistically significant difference (*p* < 0.001, r_rb_ = 0.21) (Table 1).

A total of 37 patients (~5%) received perioperative erythrocyte transfusions (Table 2). The most common number of erythrocyte concentrates per patient was two (29 cases, ~4% of the population, ~78% of all erythrocyte transfusions). In only two cases (~5% of all erythrocyte transfusions), one concentrate was deemed to be sufficient. Overall, 89 erythrocyte concentrates were transfused over the timeframe of this study. Most of the erythrocyte concentrates were needed for the THR group (n = 71; 80%) while 18 erythrocyte concentrates (20%) were needed for the TKR group. In the total knee replacement procedures, a tourniquet was always used during cementing. The THR procedures required significantly more erythrocyte transfusions (8%) in comparison with the TKR procedures (2%) (*p* < 0.001), and the relative risk was 4 times higher (RR = 4.10).

**Figure 3:**
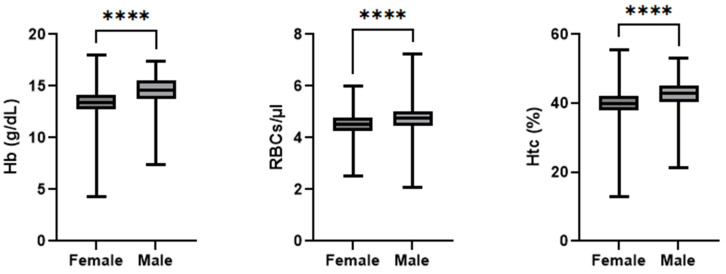
Hemoglobin levels of female and male patients at admission. **** indicates statistically significant difference between groups (*p* < 0.0001).

Splitting the patient collective into three distinct groups based on the length of hospital stay revealed a markedly higher transfusion rate in cases with longer-than-usual hospital stays. Group 1 (4–8-day hospital stays) consisted of 559 patients who stayed for the expected number of days or even slightly fewer days than the average. Group 2 (9–17 days) comprised 223 patients who needed longer than usual to recover from their operation and group 3 (>17 days) consisted of 19 patients with extremely long hospital stays. Since hemoglobin is the main parameter in the diagnosis and investigation of anemia, it deserves a particularly thorough examination. Using the Shapiro–Wilk Test, the following variables showed a difference in comparison with a normal distribution: the RBC count and Hb and Hct levels at admission for both genders (all *p* < 0.001). Due to the gender-specific differences in hemoglobin standard values, all the hemoglobin-related data for the female and male patients are shown separately (*p* < 0.001, r_rb_ = 0.51). Figure 3 depicts a summary of the admission hemoglobin data. The female patients’ hemoglobin levels (mean: 13.3 g/dL; median: 13.4 g/dL; IQR: 1.4 g/dL; min: 4.3 g/dL; max: 18.0 g/dL) were, on average, 1.15 g/dL lower than of males (mean: 14.5 g/dL; median: 14.6 g/dL; IQR: 1.8 g/dL; min: 7.4 g/dL; max: 17.4 g/dL). Notably, one female patient entered the hospital with a hemoglobin concentration of 4.34 g/dL, which is rather low. This value was double-checked during data collection and was confirmed to be the value recorded in the patient records. This testing was performed in a collaborative laboratory and not in the hospital itself, and thus, further information was not available. In total, two patients were admitted with hemoglobin concentrations below the generally acknowledged transfusion trigger of 8 g/dL. The RBC count at admission was statistically significant lower (*p* < 0.001 r_rb_ = 0.29) in the female patients (mean: 4.5 cells/µL; median: 4.5 cells/µL; IQR: 0.6 cells/µL; min: 2.5 cells/µL; max: 6.0 cells/µL) than the male patients (mean: 4.7 cells/µL; median: 4.7 cells/µL; IQR: 0.5 cells/µL; min: 2.0 cells/µL; max: 7.2 cells/µL). The Hct level at admission was statistically significant lower (*p* < 0.001, r_rb_ = 0.41) in the female patients (mean: 40%; median: 40%; IQR: 4%; min: 135; max: 56%) than the male patients (mean: 425; median: 43%; IQR: 5%; min: 215; max: 53%).

**Figure 4:**
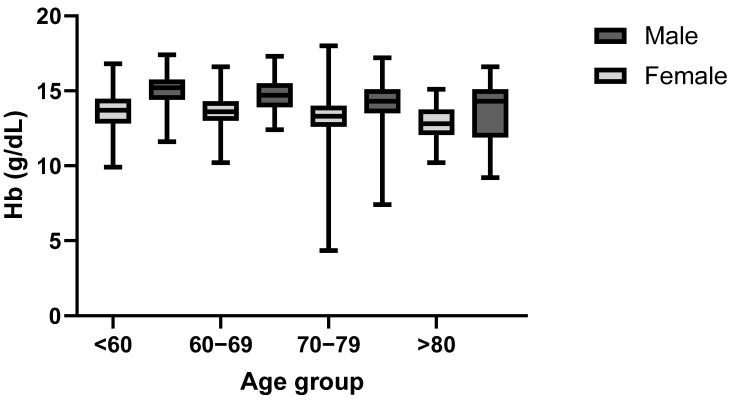
Hemoglobin level at admission in male and female patients by age group.

When analyzing the Hb levels of the groups based on gender and age, a normal distribution was found for the female patients < 60 (*p* = 0.178) and >80 (*p* = 0.679) years old, while the data was not normally distributed for the female patients that were 60–69 (*p* = 0.005) and 70–79 (*p* < 0.001) years old. A normal data distribution was found for male patients < 60 (*p* = 0.40), 60–69 (*p* = 0.682), and >80 (*p* = 0.079) years old, while between 70 and 79 years, the data was not normally distributed (*p* < 0.001). Comparing the age groups of the same gender (see Figure 4) suggested a greater decline in mean hemoglobin concentration in the aging male population (*p* < 0.001, ε^2^ = 0.07), whereas the female patients’ hemoglobin levels (*p* < 0.001, ε^2^ = 0.05) stayed much more consistent at older ages. Furthermore, among individuals aged 80 years or older (female and male), the 25th percentile had Hb levels below the WHO’s non-anemic diagnostic threshold (12 g/dL for non-pregnant female adults and 13 g/dL for male adults). A statistically significant difference was found in the pairwise comparison of the <60 and >80 age groups (both *p* = 0.001, female r = 0.33, male r = 0.39), <60 and 70–79 (female *p* = 0.042, r = 0.16; male *p* = 0.001, r = 0.29), and 60–69 and >80 (female *p* = 0.001, r = 0.30; male *p* = 0.045, r = 0.23). Additionally, a statistically significant difference was found between the 60–69 and 70–79 age groups (*p* = 0.015, r = 0.17) for the female patients.

**Figure 5:**
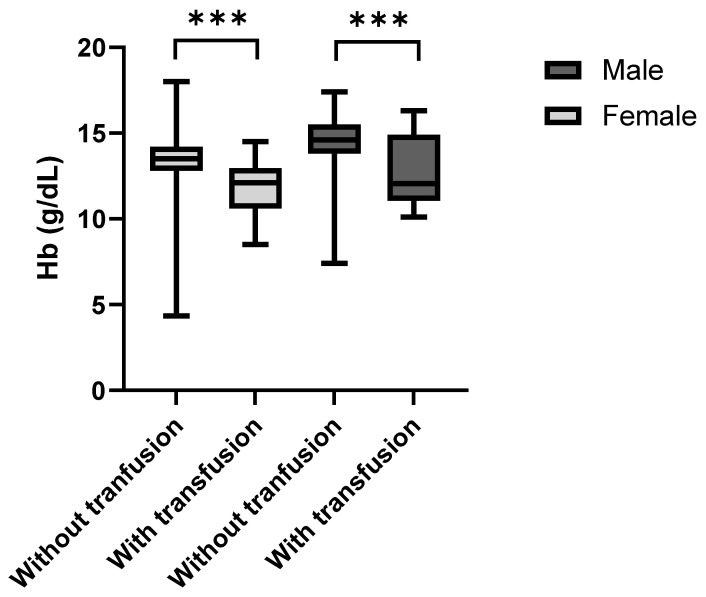
Hb levels of transfused vs. non-transfused female and male patients at admission. *** indicates statistically significant difference between groups (*p* < 0.0002).

The Kolmogorov–Smirnov Test was used to assess the data distribution of the male and female patients who received or did not receive a blood transfusion as the sample size was small for the group that received blood transfusions. The data was normally distributed for the patients that received a blood transfusion (both *p* > 0.001) but not for the patients that did not receive a blood transfusion (*p* < 0.001). The patients that received blood transfusions after surgery showed noticeably lower admission (preoperative) hemoglobin concentrations than the non-transfused individuals (Figure 5). This was the case for both female (*p* < 0.001, r_rb_ = 0.62) and male patients (*p* = 0.009, r_rb_ = 0.52). The median preoperative hemoglobin deficit among females was 1.4 g/dL and 2.5 g/dL among males.

**Figure 6:**
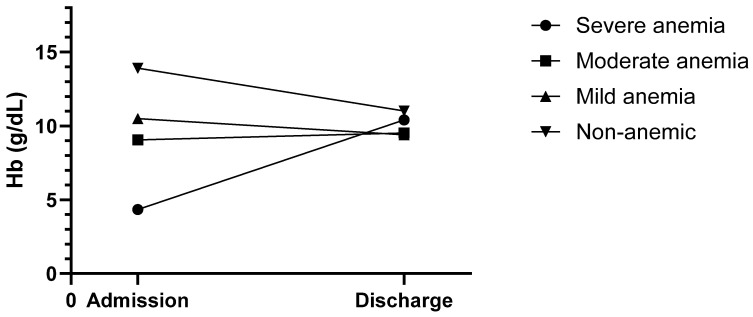
Changes in mean hemoglobin levels over course of hospital stay.

At discharge, the median hemoglobin levels decreased by 2.70 g/dL to 10.6 g/dL (*p* < 0.001 r_rb_ = 1.01) in the female patients (mean: 10.67 g/dL; median: 10.6 g/dL; IQR: 1.8 g/dL; min: 7.1 g/dL; max: 15.0 g/dL) and by 3.00 g/dL to 11.5 g/dL (*p* < 0.001, r_rb_ = 1.01) in the male patients (mean: 11.44 g/dL; median: 11.5 g/dL; IQR: 2.0 g/dL; min: 7.9 g/dL; max: 17.1 g/dL). The change in mean hemoglobin levels over the course of the hospital stay, categorized by the mean admission hemoglobin concentration, is shown in Figure 6. The funnel-like shape of the graph clearly indicates that the hemoglobin concentrations started to converge into a much narrower range over the course of the hospital stay until discharge. Furthermore, the severe and moderate anemia groups showed a rise in hemoglobin levels, with all groups being above the suggested transfusion trigger of 8 g/dL.

**Figure 7:**
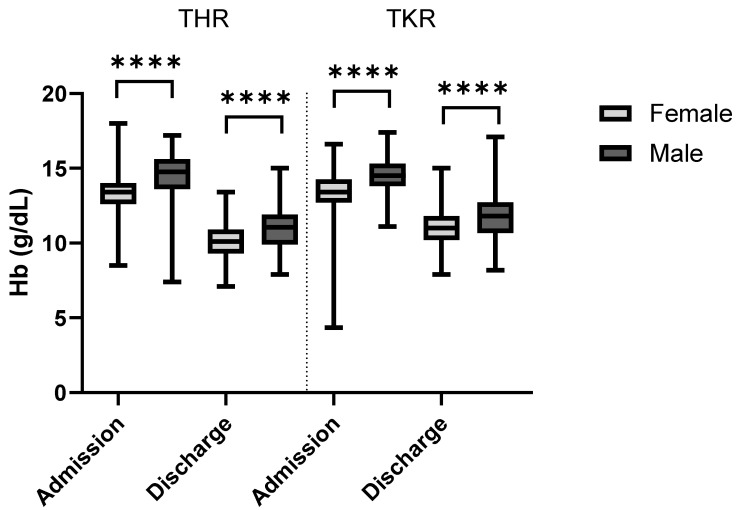
Boxplot of Hb levels at admission and discharge by procedure and gender. **** indicates statistically significant difference between groups (*p* < 0.001).

The changes in Hb levels were significantly different in comparison with a normal distribution for the THR (*p* = 0.002) and TKR (*p* < 0.001) groups, and female (*p* < 0.001) and male (*p* = 0.006) patients according to the Shapiro–Wilk Test. The changes in Hct levels were not normally distributed for the THR (*p* = 0.001) and TKR (*p* < 0.001) groups, female (*p* < 0.001) and male (*p* = 0.004) patients, and those that did not receive a transfusion (*p* < 0.001); the Hb levels in the patients who received a transfusion showed a normal distribution (*p* = 0.109). The changes in RBC counts were not normally distributed for the THR (*p* = 0.001) and TKR (*p* < 0.001) groups, female (*p* < 0.001) and male (*p* < 0.001) patients, and those that did (*p* = 0.036) and did not receive a transfusion (*p* < 0.001). Figure 7 shows the admission and discharge hemoglobin values for both sexes and procedures. As expected, the overall hemoglobin levels were markedly lower at discharge than admission. Within the study population, total hip replacement surgery was associated with a significantly higher hemoglobin change (−3.3 g/dL) than TKR (−2.5 g/dL; *p* < 0.001, r_rb_ = 0.36). The hemoglobin values of the female patients dropped significantly less (−2.7 g/dL) than those of the male patients (−3.1 g/dL; *p* < 0.001, r_rb_ = 0.15). It was also observed that with increasing age, the average hemoglobin changes remained the same (<60: −2.9 g/dL; 60–70: −2.9 g/dL; 70–80: −2.8 g/dL; >80: −2.6 g/dL) (*p* = 0.471), which may be due to the lower initial hemoglobin levels. Regarding hospital LOS, there was no statistically significant difference (*p* = 0.744) in the changes in hemoglobin level between patients staying > 17 days (−2.6 g/dL), 9–17 days (−9 g/dL), and 4–8 days (−2.8 g/dL). Lastly, the administration of erythrocyte concentrates was, as expected, associated with a significantly smaller hemoglobin decrease (−2.9 g/dL in non-transfused patients vs. −2.2 g/dL in transfused individuals; *p* = 0.005, r_rb_ = 0.27). For further information including 95% confidence intervals, see Table 3.

When analyzing the changes in Hb levels in each age group, a normal distribution was found for the 60–69 (*p* = 0.416) and >80 (*p* = 0.291) groups but not the <60 (*p* = 0.037) and 70–79 (*p* < 0.001) groups. When analyzing the changes in RBC counts, a normal distribution was found for the >80 (*p* = 0.322) age group but not the <60 (*p* = 0.007), 60–69 (*p* = 0.024), and 70–79 (*p* < 0.001) groups. When analyzing the changes in Hct levels, a normal distribution was found for the <60 (*p* = 0.152), 60–69 (*p* = 0.770), and >80 (*p* = 0.583) age groups but not the 70–79 (*p* < 0.001) group. The changes in Hb levels in the different LOS groups showed a normal distribution for the patients with an LOS > 17 days (*p* = 0.076) but not those with an LOS of 4–8 days (*p* < 0.001) or 9–17 days (*p* < 0.001). The changes in RBC counts showed a normal distribution for the patients with an LOS > 17 days (*p* = 0.059) and 9–17 days (*p* = 0.106) but not those with an LOS of 4–8 days (*p* < 0.001). The changes in Hct levels showed a normal distribution for the patients with an LOS > 17 days (*p* = 0.496) but not those with an LOS of 4–8 or 9–17 days (both *p* < 0.001).

Between admission and discharge, the TKR group showed smaller changes in RBC count and Htc level than the THR group (RBC count: −1.1 vs. −0.8 cells/µL, *p* < 0.001, r_rb_ = 0.35; Htc level: −7 vs. −10%, *p* < 0.001, r_rb_ = 0.36). Regarding gender differences, the female patients showed significantly smaller changes in RBC count and Htc level in comparison with the male patients (RBC count: −0.9 vs. 1.0 cells/µL, *p* = 0.018, r_rb_ = 0.10; Htc level: −8 vs. −9%, *p* = 0.006, r_rb_ = 0.11). No statistically significant difference in changes in RBC counts and Htc levels were found when dividing the patients by age group or LOS (RBC count: age *p* = 0.923, LOS *p* = 0.566; Htc level: age *p* = 0.564, LOS *p* = 0.760). The patients that received a transfusion showed significantly smaller changes in RBC count and Htc level in comparison with the patients that did not receive a transfusion (RBC count: −2.2 vs. −2.9 cells/µL, *p* = 0.004, r_rb_ = 0.28; Htc level: −6 vs. −9%, *p* = 0.001, r_rb_ = 0.33).

Upon admission to the hospital, a not inconsiderable number of patients (3%) were already suffering from some form of anemia (4% of the female and 1% of the male patients). Interestingly, there was a considerable discrepancy in the number of cases of mild and moderate anemia, as 1% of the male patients but 4% of the female patients (an almost 4-fold difference) were admitted with low hemoglobin concentrations (7–10.9 g/dL for females and males).

**Figure 8:**
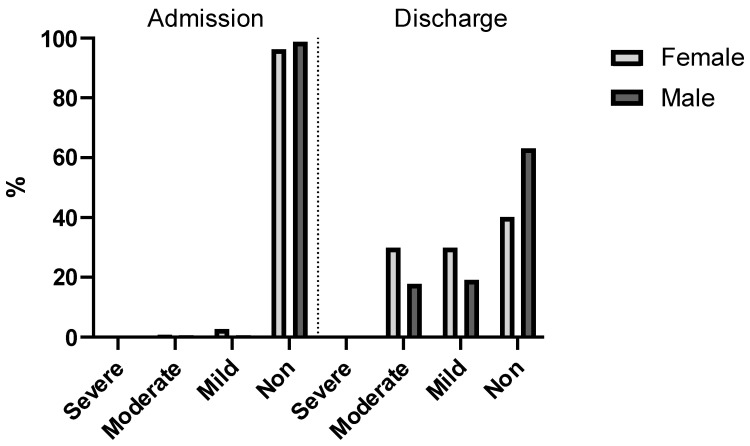
Anemia severity at admission and discharge divided by gender. The dotted line separates HB levels at admission and discharge.

Figure 8 shows after the operation was performed and before discharge, the hemoglobin levels were significantly lower, as expected (*p* < 0.001, V = 0.77). At this point, non-anemic patients were no longer in the majority (merely 49%), and moderately and mildly anemic patients comprised 51%. This was also the case among female patients (60%). Among the male population, most of the patients (47%) had mild or moderate anemia after surgery.

## 4. Discussion

“PBM is a patient-centered, systematic, evidence-based approach to improve patient outcomes by managing and preserving a patient’s own blood, while promoting patient safety and empowerment” [9,22].

**Figure 9:**
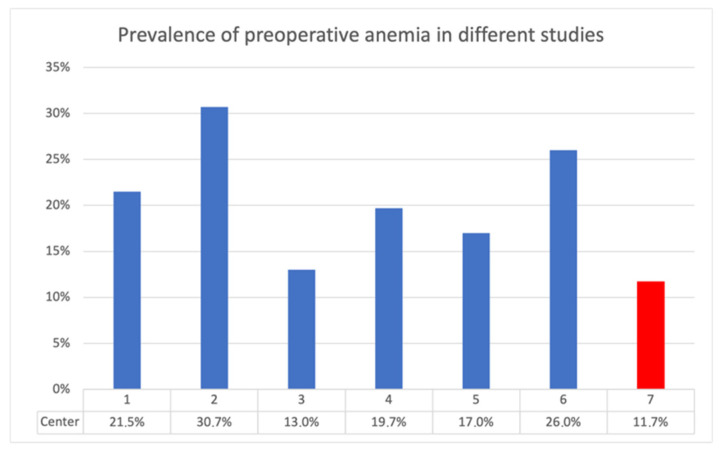
Prevalence of preoperative anemia in different studies.

Compared with other studies on the topic, this study, using data acquired at the University Hospital Krems, showed lower preoperative anemia rates. In some instances, the anemia prevalence was even less than half (Figure 9). This is evidence of good implementation of patient blood management. “It is, however, unclear whether preoperative anemia increases risk or reflects ongoing burden of comorbidities in the patient” [9]. “Most of the patients scheduled for THR and TKR are elderly, and commonly have associated medical problems like heart disease or coexisting organ dysfunction, which can compromise their outcome after the procedure” [42]. Similar to other studies in the literature, the patients undergoing joint replacement in this study were elderly. The largest age group was 70–80 years. The THR group required significantly more erythrocyte transfusions (8%) in comparison with the TKR group (2%) (*p* < 0.001), with a 4-fold higher relative risk (RR = 4.10).

**Figure 10:**
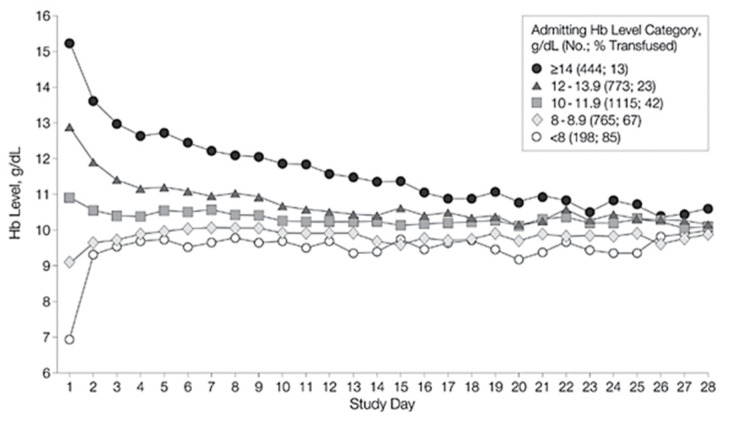
Hb convergence from Vincent, J. L., et al. (2002) [43] (“Anemia and blood transfusion in critically ill patients.” *JAMA* 288(12): 1499–1507).

Unfortunately, recent data on this topic from a surgical point of view are scarce. Therefore, the comparability of the data sets might be poor due to the use of old studies. Detailed descriptions of the various studies are provided in Appendix A. In 2002, Vincent et al. studied the changes in hemoglobin levels over patients’ hospital stay [43]. Their sample included 1136 patients from 145 different intensive care units, all situated within Western Europe. Hemoglobin data were collected daily for up to 28 days or until discharge. The patients presented vastly different hemoglobin values at admission but upon discharge, the hemoglobin values started to converge, nearing a value of around 10 g/dL [43] (Figure 10).

**Figure 11:**
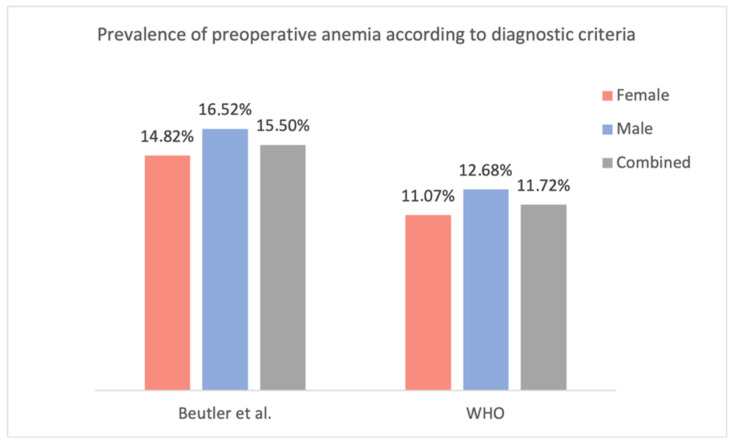
Prevalence of preoperative anemia according to diagnostic criteria [8,44].

Of particular interest is the fact that the levels of the “8–8.9 g/dL” admitting Hb group started to rise as well. Considering the transfusion trigger of 8 g/dL, this should not have happened. Stimulated by these results, a similar but much simpler evaluation was performed in this study. A rise in hemoglobin level in the groups above the transfusion trigger was noticed as well. However, it should be noted that the increase was subtle and only affected a group consisting of a single patient. In their 2006 study published in *Blood*, Beutler et al. challenged the traditional WHO criteria for anemia, which are used to define anemia in most studies, including this one. Utilizing data from the third (US) National Health and Nutrition Examination Survey (NHANES-III) and the Scripps–Kaiser database (San Diego Area, CA, USA), they proposed new lower limits for normal hemoglobin concentrations for black and white adults [44]. When evaluating the present study population using the lower limits of the normal Hb concentration proposed by Beutler et al., the prevalence of preoperative anemia increases to 14.82% in females (vs. 11.07% according to the WHO criteria; +3.75%) and 16.52% in males (vs. 12.68%; +3.84%) (Figure 11). Unfortunately, as Beutler et al. only proposed ‘lower limits of normal’ and did not specify additional subgroups (mild, moderate, etc.), only their criteria for the presence of anemia could be compared with the WHO’s criteria.

**Figure 12:**
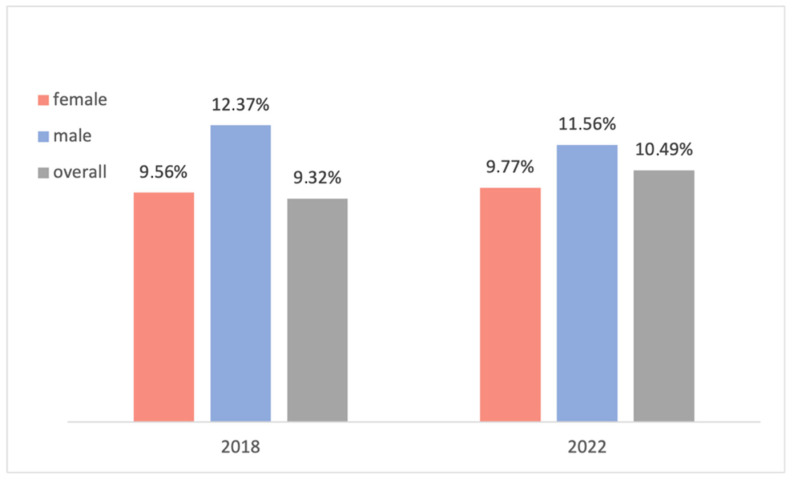
Comparison of preoperative anemia incidence in this study and previous studies.

A closer look at the transfused patients revealed a potential tendency towards double-unit transfusion. This is reflected in the frequent occurrence of transfusion of two red cell concentrates per patient and in the fact that the number of transfused red cell concentrates was almost always a multiple of two (with only three exceptions). This seems to be contrary to recommended clinical practice, as there is no scientific basis for the superiority of double-unit transfusion. Therefore, prioritization of a single-unit transfusion regime and gradual controlled substitution of the missing erythrocyte volume until the recommended transfusion trigger is reached could avoid unnecessary transfusions and reduce costs and transfusion risks [5,6,19,45]. Further studies could be performed to investigate this suspicion in depth. In 2018, a similar but more superficial data analysis was performed on a comparable population of 233 patients undergoing elective orthopedic endoprosthesis surgery. In that study, the overall prevalence of preoperative anemia was 9.32%, and 9.56% and 12.37% for female and male patients, respectively. Only the WHO criteria were utilized to diagnose anemia, and reoperations were excluded. These values are only marginally different from the population in the current study at 10.49% (+1.17%) for the overall population, 9.77% (+0.21%) for female patients, and 11.56% (−0.81%) for male patients (population without reoperations) (Figure 12). Note that to accurately compare the results, the patients who underwent a reoperation were excluded from the compared population, and hence the different values in the 2022 study in Figure 12.

The retrospective study design of this study means that some laboratory analyses were performed outside the clinic; consequently, there is no information on the test standardization, which could affect the reliability of the data. However, each report had the reference range that the preoperative anemia diagnosis was based on. Regarding the data collection, many lab values were available but were not analyzed in this study as these tests were only performed in a very limited number of patients. There is also reason to believe that in some cases, a transfusion could have been administered without a note in the discharge report. This could not be analyzed by the retrospective study design as all transfused units are registered in the clinic’s internal registry but verification was technically impossible. The discharge report was a major source for the data collection (besides lab reports), which could result in the calculated transfusion rate underestimating the actual transfusion rate.

Furthermore, in some cases, it was apparent that the discharge report was missing diagnoses of internal ailments that were either made during an internal medicine council within the hospital, during the preoperative internal assessment, or were an already known comorbidity. If relevant diagnoses from the preoperative internal assessment were not included in the discharge letter by the treating physicians, it could limit the completeness of the data collected.

Had more measurement time points been included in the study design, a much more detailed description of the hemoglobin changes during hospitalization would have been possible. However, since such frequent measurements are not part of everyday clinical practice, a prospective study design would be needed.

Finally, a follow-up study of the discharged patients would have been interesting to assess individual anemia recovery (with respect to nadir Hb concentration). But this too would require a much more complex study design.

## 5. Conclusions

Preoperative anemia can have various causes and must be specifically treated before elective surgeries as it is a modifiable factor that affects the postoperative complication rate and mortality. Preoperative treatment of anemia can increase the Hb level to such an extent that transfusions can be avoided.

However, most national and international guidelines do not account for sex-specific transfusion triggers or transfusion volumes. Hemostatic drugs such as tranexamic acid (an antifibrinolytic) and desmopressin (a von Willebrand factor-releasing drug) have also shown effectiveness in hemorrhage prophylaxis. As recent developments during the COVID-19 pandemic showed, blood transfusions and the availability of blood products are still precarious topics. In the present retrospective study that surveyed anemia prevalence and incidence in the setting of elective orthopedic surgery, the data obtained was satisfactory. In comparison with other studies (in Austrian hospitals and worldwide), the gathered data suggests superior implementation of patient blood management. With the increase in joint arthroplasties, surgeons take this issue into consideration.

## Figures and Tables

**Table 1:** Demographic data of the study population (IQR: interquartile range).

Item	m	THR	TKR
(N)	801	354 (42%)	447 (56%)
Sex			
Female	481 (60%)	200 (56%)	281 (63%)
Male	320 (40%)	154 (44%)	166 (37%9
Age (Median)	70 (IQR 15)	70 (IQR 15)	70 (IQR 14)
Age			
<50	27 (3%)	13 (4%)	14 (3%)
50–60	130 (16%)	60 (17%)	70 (16%)
60–70	226 (28%)	102 (29%)	124 (28%)
70–80	324 (40%)	137 (39%)	187 40%)
80–90	94 (12%)	42 (12%)	52 (12%)
Hospital Length of Stay			
4–8	559 (70%)	262 (74%)	297 (66%)
9–17	223 (28%)	83 (23%)	140 (31%)
>17	19 (2%)	9 (3%)	10 (2%)
ICU Length of Stay			
0	798 (99%)	352 (99%)	446 (99%)
2	1 (<1%)	1 (<1%)	.
4	1 (<1%)	.	1 (<1%)
12	1 (<1%)	1 (<1%)	.

**Table 2:** Transfused erythrocyte concentrates.

Number of Transfused Erythrocyte Concentrates	% (N)	THR % (N)	TKR %(N)
0	95.4% (764)	92.1% (326)	98.0% (437)
1	0.2% (2)	0.6% (2)	
2	3.6% (29)	5.6% (20)	2.0% (9)
3	0.1% (1)	0.3% (1)	
4	0.4% (3)	0.8% (3)	
6	0.1% (1)	0.3% (1)	
8	0.1% (1)	0.3% (1)	

**Table 3:** Change in Hb level, RBC count, and Htc level (discharge-admission) (mean [95% CI]).

Procedure	Change in Hb (g/dL)	*p*-Value	Change in RBCs (cells/µL)	*p*-Value	Change in Htc (%)	*p*-Value
THR	−3.3 [−3.4; −3.2]	<0.001	−1.1 [−1.1; −1.0]	<0.001	−10 [−10; −9]	<0.001
TKR	−2.5 [−2.6; −2.3]		−0.8 [−0.9; −0.8]		−7 [−8; −7]	
Sex	Change in Hb (g/dL)	*p*-Value	Change in RBCs (cells/µL)	*p*-Value	Change in Htc (%)	*p*-Value
Female	−2.7 [−2.8; −2.5]	<0.001	−0.9 [−0.9; −0.8]	0.018	−8 [−9; −8]	0.006
Male	−3.1 [−3.2; −2.9]		−1.0 [−1.0; −0.9]		−9 [−10; −9]	
Age	Change in Hb (g/dL)	*p*-Value	Change in RBCs (cells/µL)	*p*-Value	Change in Htc (%)	*p*-Value
<60	−2.9 [−3.1; −2.7]	0.471	−1.0 [−1.0; −0.9	0.923	−9 [−9; −8]	0.564
60–70	−2.9 [−3.1; −2.7]		−0.9 [−1.0; −0.9]		−9 [−9; −8]	
70–80	−2.8 [−3.0; −2.7]		−0.9 [−1.0; −0.9		−9 [−9; −8]	
>80	−2.6 [−3.0; −2.3]		−0.9 [−1.0; −0.8]		−8 [−9; −7]	
LOS	Change in Hb (g/dL)	*p*-Value	Change in RBCs (cells/µL)	*p*-Value	Change in Htc (%)	*p*-Value
4–8	−2.8 [−2.9; −2.7]	0.744	−0.9 [−1.0; −0.9]	0.566	−9 [−9; −8]	0.760
9–17	−2.9 [−3.1; −2.7]		−1.0 [−1.0; −0.9]		−9 [−9; −8]	
>17	−2.6 [−3.4; −1.8]		−0.9 [−1.1; −0.6		−8 [−10; −5]	
Ery	Change in Hb (g/dL)	*p*-Value	Change in RBCs (cells/µL)	*p*-Value	Change in Htc (%)	*p*-Value
No transfusion	−2.9 [−3.0; −2.8]	0.005	−0.9 [−1.0; −0.9]	0.004	−9 [−9; −8]	0.001
Received transfusion	−2.2 [−2.8; −1.7]		−0.7 [−0.9; −0.5]		−6 [−8; −5]	

## Data Availability

The original contributions presented in this study are included in the article. Further inquiries can be directed to the corresponding author(s).

## Appendix A. Studies Compared with the Present Study

The following table provides additional information on the studies compared to this study. Relevant studies were found using the PubMed tool of the US National Library of Medicine. The search terms were “preoperative” AND “anemia” AND “elective orthopedic surgery”.

**Table A1:** Studies compared with present study.

Study	Prevalence	N	Criteria	First Author and Year	Period	Country/Region
1	21.5%	881	WHO	Wan, 2020	2016–2018	Sweden
2	30.7%	234	WHO	Duarte, 2021	18 January–20 July	Brazil
3	13.0%	1335	WHO	Lasocki, 2015	10 November–11 March	Europe
4	19.7%	7975	WHO	Meybohm, 2020	12 January–18 September	Germany
5	17.0%	100	WHO	Kearney, 2015	10 March–13 June	Australia
6	26.0%	1286	<13	Munoz, 2017	8 January–14 December	Spain
*7*	11.7%	845	WHO	present study	18 July–19 December	Austria

Studies three and four included operations other than hip and knee endoprosthesis surgeries but they reported the prevalence rate for each type of operation; the combined prevalence of knee and hip endoprosthesis surgeries was calculated and used in the comparison.
